# Condition Monitoring of Additively Manufactured Injection Mould Tooling: A Review of Demands, Opportunities and Potential Strategies

**DOI:** 10.3390/s23042313

**Published:** 2023-02-19

**Authors:** Albert Weinert, David Tormey, Christopher O’Hara, Marion McAfee

**Affiliations:** 1 Centre for Mathematical Modelling and Intelligent Systems for Health and Environment (MISHE), Atlantic Technological University, ATU Sligo, Ash Lane, F91 YW50 Sligo, Ireland; 2 Centre for Precision Engineering, Materials and Manufacturing (PEM Centre), Atlantic Technological University, ATU Sligo, Ash Lane, F91 YW50 Sligo, Ireland; 3 I-Form SFI Research Centre for Advanced Manufacturing, Atlantic Technological University, ATU Sligo, Ash Lane, F91 YW50 Sligo, Ireland

**Keywords:** tool condition monitoring, additive manufacturing, injection mould tooling, structural health monitoring, acoustic emission, accelerometer, ultrasound, fault detection

## Abstract

Injection moulding (IM) is an important industrial process, known to be the most used plastic formation technique. Demand for faster cycle times and higher product customisation is driving interest in additive manufacturing (AM) as a new method for mould tool manufacturing. The use of AM offers advantages such as greater design flexibility and conformal cooling of components to reduce cycle times and increase product precision. However, shortcomings of metal additive manufacturing, such as porosity and residual stresses, introduce uncertainties about the reliability and longevity of AM tooling. The injection moulding process relies on high volumes of produced parts and a minimal amount of tool failures. This paper reviews the demands for tool condition monitoring systems for AM-manufactured mould tools; although tool failures in conventionally manufactured tooling are rare, they do occur, usually due to cracking, deflection, and channel blockages. However, due to the limitations of the AM process, metal 3D-printed mould tools are susceptible to failures due to cracking, delamination and deformation. Due to their success in other fields, acoustic emission, accelerometers and ultrasound sensors offer the greatest potential in mould tool condition monitoring. Due to the noisy machine environment, sophisticated signal processing and decision-making algorithms are required to prevent false alarms or the missing of warning signals. This review outlines the state of the art in signal decomposition and both data- and model-based approaches to determination of the current state of the tool, and how these can be employed for IM tool condition monitoring. The development of such a system would help to ensure greater industrial uptake of additive manufacturing of injection mould tooling, by increasing confidence in the technology, further improving the efficiency and productivity of the sector.

## 1. Introduction

**Figure 1:**
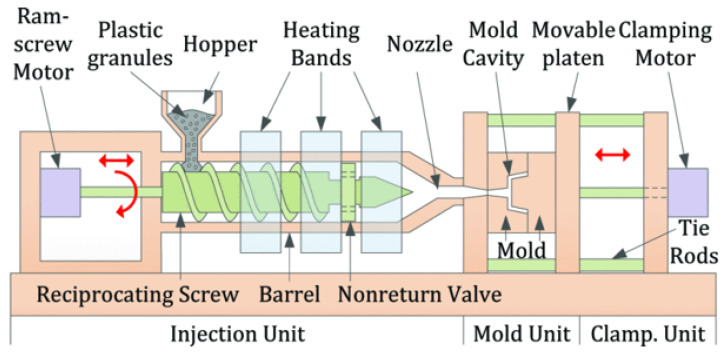
Depiction of an injection moulding machine and mould. Adapted with permission from Ref. [2], Copyright (2019), IEEE.

Injection moulding (IM) is commonly used to fabricate products rapidly and at high volumes from plastic pellets. Some items produced through this process are toys, automotive body parts, medical and pharmaceutical devices, etc. Around 30% of plastic parts used daily are manufactured through the IM process [1]. The moulded parts emerge in a final form with no or minimal post-processing required. The mould tool is inserted into the injection moulding machine (IMM) and clamped between a moving and stationary platen as shown in Figure 1.

The mould tool is connected to the machine platens to form the moulded components within a closed mould cavity. After forming, the mould tool opens and ejects the moulded components. Mould tools consist of the cavity, cooling channels, ejector pins and other features such as locations for sensors. With all these components present in the mould, the available space is very limited. Therefore, highly skilled tool designers are needed to design both halves of the tool, containing all the geometry and features required to address the specification of the final moulded part.

The tooling is very complex to manufacture. This mainly consists of milling operations to cut out the required slots and shapes for ejection pins, cooling channels, entry gates, sensor slots and other additional features to be incorporated [3].

The location of cooling channels has an essential influence on the solidification of the part. Cooling channels are bored in each half of the mould and used to cool the polymer inside the mould using water [4]. The cooling channels are usually located as close as possible to the cavity to accelerate the cooling stage of the process. Conventional cooling channels are typically straight as they are made using drilling operations. The geometry of injection moulded parts may be intricate, and thus conventional cooling channels may not be located as close to the cavity walls as is desired. The cavity walls must withstand the pressures applied to the mould during the injection stage and the water pressure passing through the cooling channels. This limits the minimum distance between the cooling channels and the cavity wall due to the increased potential for tool failure with thinner walls. If the wall thickness separating the cooling channels and cavity is too small, tool fatigue can cause a crack resulting in potential water leakage into the polymer. Any potential faults occurring in the tool result in catastrophic failure not only for the tool but also can result in significant damage to the machine.

These complexities in the design and manufacture of injection mould tools result in long lead times and limitations in tool performance in terms of the cooling of the part. The IM industry demands faster tool design, shorter lead times for tooling, shorter production cycle times and more accurate part dimensions with low variance on part tolerances. The technology with the potential to address all of these requirements is additive manufacturing.

**Figure 2:**
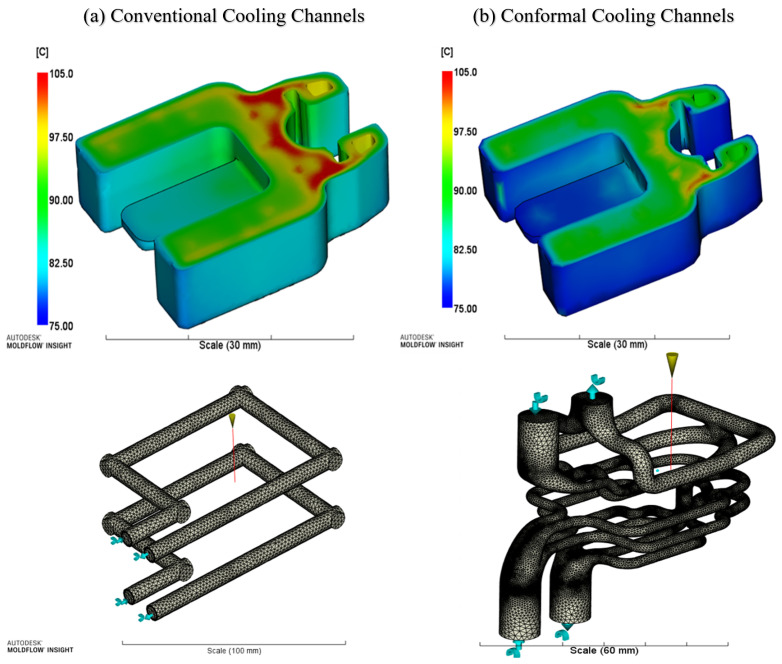
(**a**) Temperature profiles for a moulded component using conventional vs. (**b**) conformal cooling and their corresponding 3D meshed cooling desings [10].

AM processes produce physical objects from digital information piece-by-piece, line-by-line, surface-by-surface or layer-by-layer [5]. This simultaneously defines the object’s geometry and determines its material properties. AM processes place, bond and transform volumetric elements of raw material to build the final part. Each element’s shape, size and strength of the bonds between the elements are determined by the raw material(s), the manufacturing equipment’s settings, and the process parameters. AM technologies can fabricate parts without the need for intermediate shaping tools, potentially reducing the number of manufacturing steps required [6]. Additive manufacturing facilitates the manufacturing of complex internal features to increase functionality and improve the performance of injection tooling. The most significant advantage of AM over conventional machining methods for injection mould tooling is the possibility of integrating conformal cooling channels into the design of the part as it is being built layer-by-layer [7]. Conformal cooling channels can follow the external geometry of the parts, providing more effective and consistent heat transfer. AM offers faster cycle times of up to 30% [8,9] and improvements in shape quality of the parts [9] compared to conventional cavities, as shown in Figure 2.

Conformal cooling offers a new way of part tempering that could never be achieved by cooling channels produced by traditional machining methods [11]. However, with the introduction of a new manufacturing technology come certain unknowns, particularly concerns about the reliability and longevity of 3D-printed tooling. Conventional tools have been around for over 50 years, and their use has been well-established in the industry.

There are limitations associated with metal AM printed parts which need to be addressed before such methods become widely adopted for high-performance tooling. The printed parts require post-machining to address poor surface finish and residual stresses [12]. The requirement for post-machining to achieve the required surface finish means additional material should be added during the design stage, which later can be machined to bring the final parts to within the required specification [13].

Further, components manufactured by metal AM have inherent structural limitations relative to those produced by conventional machining methods. These include the presence of porosity and reduced density of printed components, which can lead to cracking and delamination [14]. As a result, attempts at adopting this new manufacturing technology into the tooling sector are slow. The risk of a mould tool failure is a major deterrent, as tool failures can significantly damage the injection moulding machine and cause significant disruption to production.

Injection mould users, therefore, require a form of monitoring the tools for safer production and a reduction in unnecessary downtimes. In other industries such as machining, aerospace and wind turbines, the application of tool condition monitoring (TCM) systems has proven to be very effective in identifying the initiation and development of defects such as cracking and delamination during operation, facilitating predictive maintenance before catastrophic failure. These systems rely on data collection during operation from embedded or attached sensors. Various approaches are taken to analyse such signals to report the health of the structure of interest.

In this paper, we propose the application of TCM for injection mould tooling to address concerns about the reliability of tools fabricated via additive manufacturing. Currently, TCM has not been used with conventional injection mould tooling; instead, tools are inspected during scheduled maintenance, requiring downtime in the process. Kek et al. [15] investigated the use of externally mounted acoustic emission sensors to differentiate between healthy and damaged conventional mould tooling in an IM process. Moreira et al. used accelerometers mounted on the injection plate for vibration monitoring in a micro-injection moulding process [16]. However, no work has been presented to date on detecting tool defects arising during operation or specifically investigating additively manufactured tooling. Opportunities for TCM are limited with conventional tooling due to the space constraints and complexities of tool manufacture. However, AM offers the potential to embed or create in situ sensors into products while they are being built [17]. Greater use of embedded sensors would significantly benefit injection mould tooling, offering enhanced monitoring of the process and in situ monitoring of the part quality and tool health. Additional sensorisation of the tooling, along with the use of TCM, can provide useful information to the user about the real-time state of the 3D-printed mould tool, offering predictive maintenance when needed, to maximise the production capacity of the tool.

This paper reviews the potential sources of defects in conventional and additively manufactured IM tooling, and the impact these defects can have on the machine, the process, and the product. Sensor technologies used in other sectors to detect similar defects are reviewed, together with a review of approaches to tool condition monitoring of moulds, tools and dies in other processes, incorporating sensors, fault detection methods and diagnostic approaches. Potential strategies to encourage the adoption of TCM in AM injection mould tooling are discussed, along with opportunities that such systems offer to impact a service model for industrial IM tooling under the paradigm of Industry 4.0.

## 2. Defects

IM tooling is designed for high-volume production, typically lasting around 1,000,000 cycles. During their lifetime, the tools are expected to withstand cyclic loading from thermal and high-pressure stresses exerted throughout the operation cycle [18]. Conventionally designed and manufactured tools have historically experienced a range of failures such as cracking, deformation, chipping, wear and cooling channel blockages which generally lead to issues associated with part quality.

This section will first provide a brief overview of defects associated with conventional tooling and introduce the additional defects that must be considered with the introduction of additive manufacturing methods for producing injection mould tooling.

### 2.1. Conventional Defects

**Figure 3:**
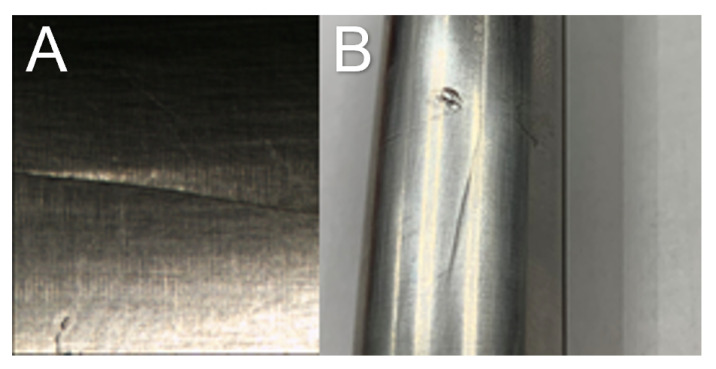
(**A**,**B**) Example of a hairline crack in an IM tool insert.

Cracking is a primary concern in injection mould tools which, depending on the location, can cause catastrophic failure. Cracks can range from hairline marks (Figure 3) which result in imprints on the produced polymer parts, to more severe failures such as crack propagation reaching the cooling channels which can cause water leakage into the cavity and lead to the early retirement of the tool [19].

**Figure 4:**
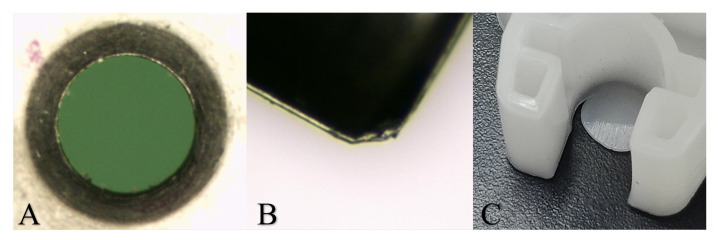
(**A**,**B**) Wear of moving parts causing the (**C**) formation of flash.

Cracking can also be caused by machining processes where sharp edges or lack of radii can cause a concentration of stresses within areas of the tool, which can initiate crack development [20]. The repeated opening/closing and ejection operation causes wear of metallic parts, which must be replaced after several thousand cycles. The wear of metallic surfaces can cause the creation of gaps through which polymer material can escape. This results in leaked polymer material forming unwanted thin layers (‘flash’) on the edges of the part at locations of the mould parting lines and vents, as shown in Figure 4 [21]. Typically, the formation of flash on manufactured components results in the rejection of components during quality checks.

**Figure 5:**
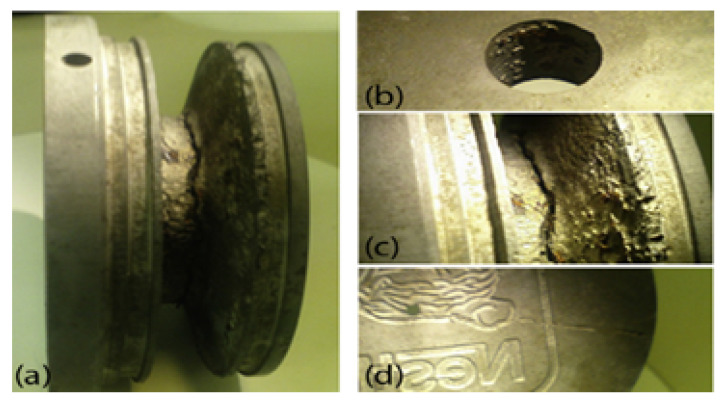
The general aspect of the side view of the die: (**a**) detail of the hole used for the assembly; (**b**) the rest of the mould; (**c**) detail of the cooling area; (**d**) detail of the mould face. Adapted with permission from Ref. [19], Copyright (2013), Elsevier.

The cooling channels in the mould are usually filled with water mixed with a solvent to reduce the formation of rust. Depending on the process environment, this solvent may not be used and the cooling channels start corroding [22], which leads to the build-up of residue and an increase in internal pressures [19]. As a result, a complex fatigue–corrosion cracking mechanism can be initiated, which can lead to damage to the tool (Figure 5). This form of blockage can lead to the development of cracks that propagate towards the surface of the cavity, causing water leakages that can mix with polymer, resulting in a catastrophic failure.

Deflection of IM tools can occur due to thin features being subjected to high pressures in the core or cavity. Deflection is an uncommon scenario within the injection moulding industry but must be considered during the tool design stage [18,23]. Such features in cavities are also known as feather edges and once deformed, they affect the manufactured components due to resulting geometric inaccuracies caused by thicker/thinner walls. These deformed features can develop into cracking, causing further damage or potentially leading to catastrophic tool failure. This typically occurs at 90° corners where excessive forces applied to the thin feature when being cyclically loaded, eventually lead to a crack formation that can potentially propagate into the tool’s structure.

Denting of the mould surface is another issue which can occur in injection mould tools; denting can also cause other defects such as cracking and delamination. Denting occurs when an unwanted material, typically polymer, becomes caught between the halves and the tool clamps the foreign body between the plates [24]. This can cause minor to severe damage depending on the nature of the foreign body and the material used for the mould. If the issue occurs in the critical region of the cavity and core used to form the final component, the tooling could become unusable, requiring further inspection to assess the damage and rectify it. Such instances will result in significant downtime in production and business losses.

### 2.2. Potential for Tool Defects Arising from Additive Manufacturing

With the introduction of metal AM into the design and manufacturing stages of injection mould tools, additional issues need to be addressed. The main concerns within the industry are the perceived lower mechanical performance of AM-produced tooling relative to conventional tooling, and the introduction of additional structural defects associated with AM [25]. Additive manufacturing consists of several processes such as liquid, molten filament, powder bed, spray powder and solid layer [26]. The most common metal AM process is selective laser sintering (SLS), where layer-by-layer a fine powder is spread and sintered to build a part. The powder is sintered using a focused laser beam generating a high amount of heat [27]. The heat generated during the process causes issues with heat transfer and the formation of residual stresses in the built parts. The build-up of residual stress occurs between the printed surface and print bed, where the stress causes compression at the centre of the build and tension at the edges, causing the components to warp [12]. When the printed part is removed from the base, some residual stress is relieved, but the parts may still deform. Typically, the parts must undergo post-processing steps such as heat treatment to relieve the stresses and avoid dimensional distortion.

**Figure 6:**
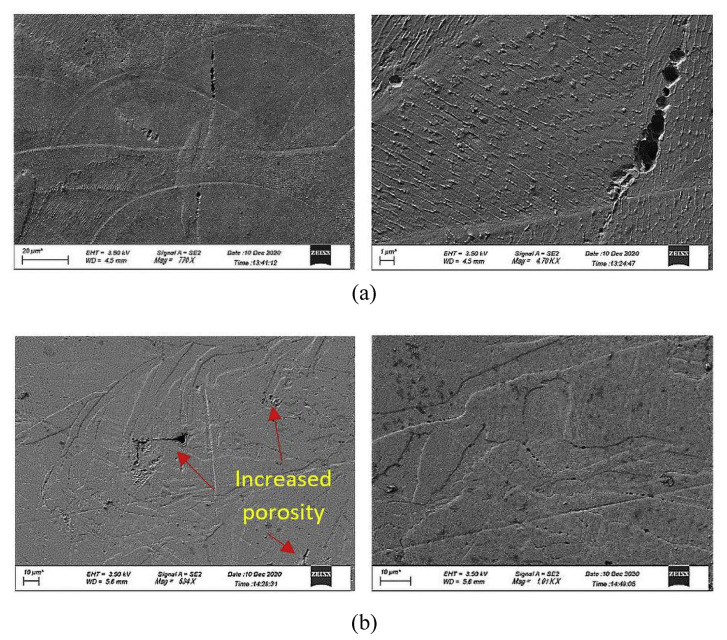
SEM micrographs of the AM samples showing the pores with samples from (**a**) EOS 280 and (**b**) Concept Laser M1. [28].

A further concern associated with SLS is the density of the printed parts. Figure 6 shows two possible cases for porosity, one being process-induced porosity, where a boundary of non-sintered powder is present between the layers, and the second is when a bubble of gas is trapped between the layers, also known as gas-induced porosity.

**Figure 7:**
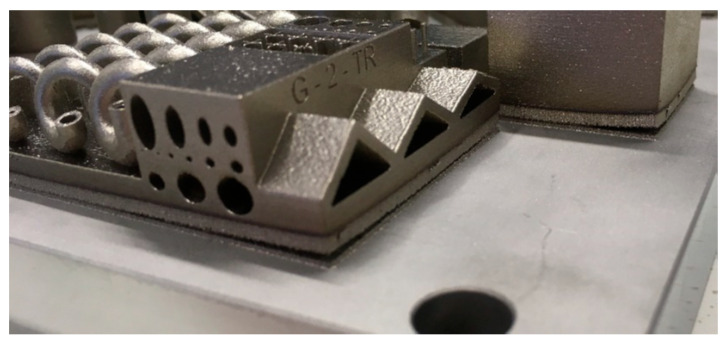
Example of layer delamination [30].

There are two main reasons for porosity caused by non-sintered powder. One is the powder not being sieved, leading to a considerable variation in the particle sizes [14]. The second is the laser power being too low, causing the particles not to melt entirely. This reduces the overall density of the parts and induces unwanted pores. Pores, when present in a mould tool under high thermal and mechanical stresses, can be an initiation point for internal cracking or delamination, creating a potential for catastrophic failure in the process due to tool failure. An example of cracking in tooling has been covered in the previous section in Figure 6. An example of layer delamination is shown in Figure 7 where two layers are debonding due to poor sintering and fusion between layers [29].

Metal additive manufacturing typically produces near-net-shape parts which require post-machining [31] as the surface finish of the 3D-printed components are still rough. An improvement in the surface finish is possible with finer powder containing finer metal particles and using refined print settings to deliver layers of a finer *z* height [13]. However, even this level of refinement is not enough for 3D-printed tooling to provide ready to mould surfaces. Consequently, the AM parts require post-machining processes such as wire or sink EDM, milling, drilling and grinding to achieve the best surface finishes typically required for the IM process. The internal surface of conformal cooling channels (CCC) also needs to be addressed, as the potential buildup of residue can cause the development of cracking in the mould tooling [19].

## 3. Sensors

Sensor technologies are used widely in machining, wind turbine and aerospace industries for monitoring structures for similar types of defects as those highlighted in Section 2.1 and Section 2.2 as risks in IM tooling (cracking, delamination, channel blocking, etc.). Sensors are placed on machine tools, wings, blades and structures to monitor their structural health and performance. If the sensors detect the structure has been worn or damaged, the components or whole assembly can be changed or repaired in time to avoid unexpected downtime and scrapped components [32]. Several different types of sensors have been applied for the early detection of defects of this nature, and the most common and practical types best suited for use in IM tooling are reviewed in this section.

### 3.1. Acoustic Emission

Acoustic emission (AE) is a well-established technology for monitoring materials under stress [33]. AE relates to the emission of strain waves in a material subjected to an external load [34]. Acoustic emission is best defined as the energy released in the material/structure at the microscale. It is derived from other types of energies, for example, vibration, force and sound [35]. Acoustic emission instruments have been developed for monitoring, and performing non-destructive testing of structural integrity for evaluating the general quality of manufacturing processes, components and devices [36]. Acoustic emission is used in tool condition monitoring (TCM) of machine tooling in processes such as milling, drilling, grinding and turning [37]. Acoustic emission sensor signals have great importance in detecting tool wear in the machining processes [35]. With a load exerted on the object of interest, defects can be identified by a change in the amplitude and frequency of acoustic emission. Chiou and Liang [38] performed an analysis of the root mean squared (RMS) AE signals from tools in a turning process. It proved to be an effective method of distinguishing between new and worn tools. Acoustic emission also detects micro-structural changes in the materials arising from fatigue cracks in wind turbine blades [39]. Mounting the sensors on the blades enables continuous monitoring of the blade health under periodical high loading [40].

Bhuiyan et al. [41,42] investigated tool condition monitoring using the acoustic emission and vibration signature in a turning process. In this study, an acoustic emission sensor and a tri-axial accelerometer were placed on the cutting tool holder’s shank to monitor the tool’s condition. Fast Fourier transform (FFT) was used to convert the captured AE and vibration signals from the time domain to the frequency domain. The raw signals and their frequency spectra were examined to identify features in the signals. It was identified that the frequency range between 68 kHz to 635 kHz was associated with chip formation during cutting. Kek et al. [15] were the first to report the use of AE for TCM of the mould tool in an injection moulding process. They compared the AE signals in healthy and damaged tool inserts during an IM process. During the trial, the damaged tool presented a higher acoustic emission amplitude. The increase resulted from the presence of microcracks in the damaged steel insert.

Acoustic emission sensors offer a nondestructive test (NDT) method for TCM. Provided there is little damping, the probe can be placed anywhere on the structure of interest, i.e., for monitoring the condition of an injection mould tool, the sensors can be placed on the outside of the mould which reduces the design complexity of fitting sensors close to the cavity.

### 3.2. Accelerometers

Detecting defects such as cracking and delamination based on vibration is performed using accelerometer sensors. The measured vibrations are referred to as displacement, velocity, or acceleration measurements. Accelerometers sense acceleration and they provide an output proportional to acceleration and vibration.

The piezoelectric accelerometer is the most common type in vibration sensing operations [43]. Accelerometers may be compression or shear types with the shear type covering compressive and impact readings. Accelerometers can provide outputs in a very small range (1 mV). They are very vulnerable to interference from the external environment and require an amplifier to increase the readability of the collected signal [44].

Accelerometers are typically used in conjunction with acoustic emission sensors for condition monitoring applications, where acoustic emission sensors capture higher frequency signals [41]. In wind turbine blades, failure events are concerned with vibration signals representing dynamic properties of the blade, such as frequency-response and modal parameters of the blade structure [40]. The sensors monitor changes in blade vibrations and detect the presence of cracking and delamination in the blade structures.

Accelerometers are also used in tool monitoring in machining operations where the captured response will vary depending on the state of the tool [38]. CNC lathes use vibration readings to monitor the health of cutting tools in heavy machining [45]. Turning [46,47] and milling [48] also have applications where using accelerometers is advantageous. They are used in monitoring the cutting tool to identify cracking or chipping on the cutting edge. Accelerometers have recently been introduced in forging processes for monitoring stamping dies during the process in TCM systems [49]. All these processes rely on high production volumes and require continuous monitoring of tools and die due to the high-value production time to meet targets, demonstrating a key advantage of implementing TCM systems. In ideal circumstances, an acoustic emission sensor should be fitted alongside an accelerometer to gather all vibration noise relevant to the tool’s state [50,51,52].

Moreira et al. [16] used an accelerometer in conjunction with custom-built pressure sensors mounted on the injection plate of a microinjection moulding machine. The collected data from the sensors provided insight into the stages of the moulding process. From the collected data, issues relevant to the moulding process can be identified such as blockages in the injection stage or ejection pin breakages.

### 3.3. Ultrasound

Ultrasonic transducers are devices that generate or sense ultrasound energy. They are divided into three categories: transmitters, receivers and transceivers. Transmitters convert electrical signals into ultrasound, receivers convert ultrasound into electrical signals, and transceivers can transmit and receive ultrasound. Depending on the application, different modes are used. They can be used in a pulse-echo mode; in this method, a transducer emits a pulse of energy and the same or a second transducer receives the reflected energy, also known as an echo. The pulse-echo method is effective when either side of the material can be used [53]. The ultrasonic waves are propagated back by the opposite face of the material or by faces resulting from separate layers, voids or inclusions in the material. These waves are then received by the same transducer, where the reflected energy is transformed into an electrical signal.

An alternative testing method is ’Through Transmission Testing’. In this method, an ultrasonic transmitter is installed on one side of the material and a receiver is installed on the opposite side [54]. This method detects, verifies and measures the growth rate of breaks in channelling, vessels, round and hollow shapes, and sometimes non-barrel-shaped vessels. Analysis of the ultrasound signals can determine the size and location of failure in adhesive joints and has been applied to detect delamination in wind turbines [55].

The pulse-echo method, using an ultrasonic transceiver, is a better method when installed in locations where space is restricted. The pulse-echo method also reduces the volume of data collected due to the collection of echoes which dissipate through the material. ’Through transmission testing’ on the other hand, offers the advantage of more data collected, but due to the need for two probes, this approach requires more installation space.

The application of ultrasound as an NDT method is very promising in the wind turbine industry [56,57]. Once an area likely to show signs of delamination is known through simulation or testing, the probe can be installed to monitor the area of concern. Soerensen et al. [58] presented work using ultrasound on wind turbine blades to identify structural delamination. This approach has been validated using X-ray to verify the presence of delamination. The ultrasound technique is being developed to identify the type of damage in wind turbine blades, such as cracking [59] and delamination, with successful attempts at localising the defects [60]. These techniques have also been introduced into the aerospace industry; due to the high demand for air travel, it is necessary to ensure all aircraft are safe before flights depart [61]. Recent advances show the successful application of this technology in this field with successful fault identification in the wings [62,63].

### 3.4. Other Sensors

Eddy Current [64] sensors have also been applied to detect surface and near-surface cracks. The occurrence of a crack can be detected by monitoring the rate of change of the current passed through the coils. Eddy current sensors have been embedded into surface washers to detect cracking at joints [65]. Detection of deflection or bending in structures is a common case in wind turbine blades and applications in aviation [66]. Strain gauges placed under the skin of the blade detect structural changes in the skin during operation and identify when a change in the structure has occurred [67]. Thermal imaging is another popular application for fault detection and has been applied in injection moulding [68]. It offers the possibility to identify surface cracking based on heat accumulation in a crack opening, as the region of interest would absorb more heat due to the increased surface area and be visible on an IR camera [69].

### 3.5. Summary

Integration of sensors into injection moulding needs to consider the limited space available. This is the primary constraint when designing suitable locations for probes in the tool. Space constraints are due to the presence of cooling channels, ejector pins, cores and cavities in more complex moulds. This can extend to servos and slides needed for complex moulding applications. This results in difficulties in specifying commercial probes, which take up space due to their size. For example, ultrasound sensors require custom-made probes, which may not be feasible or cost-effective. The use of washers or bolts can be considered for integrating eddy-current as washer sensors, but the tightening forces could cause the sensor to be damaged during installation. Placing sensors on the outer surface of the cavity inserts allows for easier integration with minimal design modifications to the tooling. They will not be directly in line with the clamping forces or the cooling channels and they are less likely to experience damage in a tool failure scenario, i.e., deformation, cracking and water damage. The main modification is conducted on the mould base itself, but the cavity inserts undergo minimal modifications.

The best-suited sensors for use in IM are AE and accelerometer sensors as they can be mounted on the external surface and do not require alignment with the region of interest. These can be mounted anywhere on the cavity and can pick up damage in every region of the cavity insert. Compared to ultrasound, they are also commercially available in smaller sizes, making them more suitable as the amount of space is limited in compact injection mould tools. Ultrasound sensors can be considered in the design but require an extended design stage for their correct operation based on location and distance from the region of interest. They also need to be aligned with the area of interest compared to AE and accelerometer.

## 4. Signal Processing

The signal processing of sensor data is essential to extract and identify information relevant to the tool condition, necessary to detect issues and schedule preventive maintenance. Effective signal processing techniques are essential for tool condition monitoring (TCM) [70] as they inform the end user of failure development and can avoid unplanned downtime in the process [71].

This section will focus on signal processing techniques commonly used in industrial processes with sensor technologies installed for condition monitoring, including acoustic emission, accelerometer, and ultrasound sensors. These sensors are the most prevalent sensors in TCM applications. Based on their application in similar industrial processes, these sensors show the greatest potential for application in injection mould AM tooling when implementing a TCM system. This section aims to provide an understanding of the primary functions achievable by using each signal processing technique and evaluate their usability in the condition monitoring of injection mould tooling.

**Figure 8:**
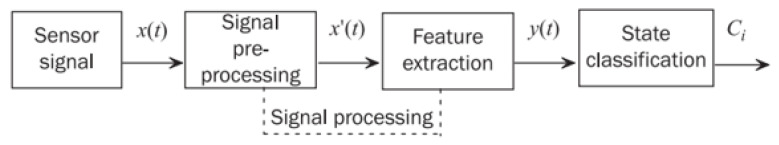
TCM as a pattern recognition system [71].

In machining processes, the most common types of signal processing techniques for TCM are Fourier transforms and wavelet analysis. The Fourier transform converts the signal from the time domain to the frequency domain. Wavelet analysis is derived from Fourier analysis and is most suitable for analysing non-stationary signals in machine tooling. Wavelet analysis enables the detection of transient changes in signal frequency content over time. Figure 8 presents a general scheme for processing and analysing sensor signals in the classification of the tool state such as healthy and defective, with the potential for identifying alternative classes of defects.

### 4.1. Fourier Transforms

Fourier transform analysis consists of several techniques, including time–frequency domain and frequency domain analysis. In time–domain analysis, the signal from the sensor is graphed with respect to time. However, there is the advantage of potentially decomposing the frequency by their source [72]. Some researchers have used time–frequency analysis to process acoustic emission signals [15] and ultrasound signals [58] for relevant condition monitoring tasks.

Noise occurs in machining processes, where mechanical interactions, such as that between bearings and shafts, generate vibrations. Each interaction is typically associated with a specific frequency, and other noise components add additional frequencies to the signal [73]. In an injection moulding process, noise can be caused by other injection moulding machines working in the background, other signal frequencies generated from the tool’s filling stage or vibrations generated by the water flowing through the cooling channels. In summary, each component or stage of the process produces a different frequency. The resulting signal read by sensors is a summation of multiple signal sources that the sensor has measured [74].

In tool condition monitoring, signals acquired from acoustic emission and accelerometer sensors deliver summed signals. Those signals typically contain information relevant to different tool conditions as well as various sources of noise [75]. With spectrum analysis, each defect may potentially be individually analysed by assessing the frequency associated with it [76,77]. Filtering using appropriate low/high pass or bandpass filters prior to sampling can be helpful in eliminating the noise from collected data and increasing the signal-to-noise ratio. However, the spectral decomposition itself may enable the isolation of a specific frequency band associated with each defect without much noise present, as typically defect signatures are of a lower frequency than noise.

By converting the signals from the time domain to the frequency domain, the user can observe a particular defect’s development with respect to an increase in the amplitude of a given frequency [78]. Novelo et al. [79] presented several analytical techniques on vibration data, i.e., Fourier transform, empirical mode decomposition, multiscale entropy (MSE), shock response spectrum and correlation to identify the fault indicators in a nut die tool. Empirical mode decomposition is a method of frequency decomposition widely used in structural health monitoring (SHM) for damage detection. The MSE technique analyses vibration signals by looking at entropy within a system to determine its health.

In a typical environment, the dies are run until catastrophic failure and then replaced; the work of Suhaimi et al. [49] aimed at predicting the failure. The captured accelerometer data were converted to the frequency domain to analyse the fault indicators. Out of the analytical tools used, the average fast Fourier transforms of the intrinsic mode functions (IMF) and correlated MSE profiles were able to identify the fault types such as quality deterioration, bending and cracking. El-Galy et al. [80] presented the feasibility of using frequency and time domain analysis on AE sensor data to detect faults in forge tooling. They used a simulated forging process, where a mathematical model of relevant faults was developed.

Based on the simulation results, the authors proposed AE pattern indicators that could distinguish between different types of defect/failure events and categorise them within the forging process.

Ket et al. [15] used acoustic emission sensors to identify cracked inserts during the filling stage of the injection moulding process. They compared AE signals from inserts known to contain cracks to AE signals generated when using healthy inserts. Higher amplitudes and energies of the measured AE signals in the defective tooling relative to the healthy set indicate the presence of cracks. The higher AE activity is caused by an increase in mechanical stresses in the injection moulding tool. Ket et al. [15] successfully analysed the AE signal using a spectrogram to evaluate the difference between a healthy and damaged tool during the injection moulding process.

**Figure 9:**
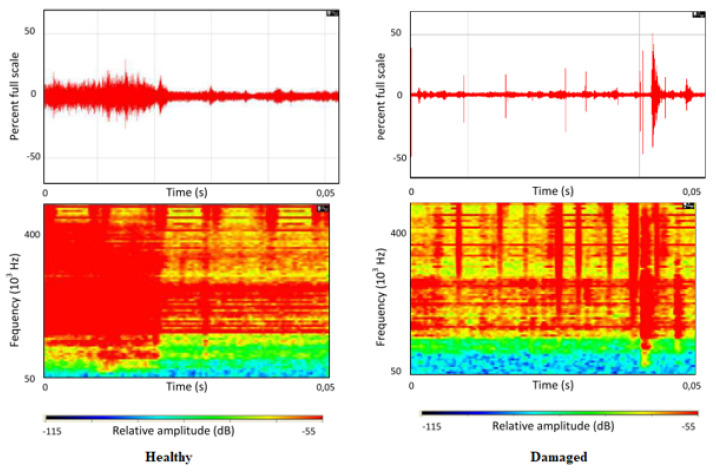
Time frequency diagram of AE signal during the filling phase of healthy and damaged mould [15].

A spectrogram is a visual representation of the range of frequencies in an AE signal as they vary with time. Spectrograms enable observation of the energy distribution of the acoustic signal derived from Fourier transforms. The time–frequency diagram’s colours represent the signal’s energy at different frequencies over a given time frame. The colour code typically uses red to represent the highest energies, with decreasing order of magnitude represented by orange, yellow, green, cyan and blue. Black areas represent frequencies below a threshold decibel level (Figure 9). As shown in the top right graph of Figure 9, the acoustic emission bursts are much more distinct in the damaged tool. These distinct red regions are indications of crack growth in the tool insert and they typically reach higher frequencies than acoustic emission bursts witnessed in the healthy tooling, as depicted in the graphs on the left.

Short-time Fourier transforms (STFT) use fixed-sized tiling; that is, segmentation of the time and frequency axes into equal-sized tiles. Once the tiling scheme or aspect ratio is defined, each cell retains an identical aspect ratio throughout the analysis. Using tiles of identical size can result in too short or too big of a window, which restricts the ability to accurately identify the low-frequency components in the signal or missing temporal changes in high-frequency components. This can result in the misidentification of failure types or, in worse cases, missing out on the defect or failure entirely due to the frequency resolution being too coarse. For example, chipping of the tool may be misidentified as a crack initiation, causing the operator to continue instead of stopping the process. Other techniques were developed and used to overcome issues of the short-time FT, such as wavelet analysis [70,72,81].

### 4.2. Wavelet Analysis

Wavelet analysis resolves resolution precision in signal processing to capture transient signals and analyse them [72], reducing the delay in identifying defects in the initial stages. Wavelets offer the advantage, relative to the STFT, of analysis with an excellent resolution for a chosen section of a signal. Wavelets offer the use of shorter time windows at high frequencies and longer time windows at lower frequencies as compared to fixed time windows for all frequency ranges in the STFT [81].

Wavelets are divided into two types of transform: continuous wavelet transforms (CWT) and discrete wavelet transforms (DWT) [70]. CWT allow for continuous analysis of a signal with a predefined wavelet. One typically assigns a wavelet with a particular event such as cracks, chipping or more severe failures and sends them continuously through the signal to find a matching amplitude and frequency to localise the failure [82]. A suitable wavelet is identified from a historical dataset representing the damage. However, in some cases, experimental test data are used where historical data are unavailable.

This form of analysis is a type of time–frequency analysis, where the signal is analysed in the frequency domain with respect to time. Each wavelet passes through the signal, essentially identifying matching signals or best matches and the point in time where they occur [83]. One main limitation of the CWT is its computational delay; as the wavelets scan through the entire signal, much of the redundant information is analysed [70]. CWT finds application in TCM and structural health monitoring (SHM), in applications such as machining processes [84,85,86] and wind turbine blades [83,87] using acoustic, ultrasonic and accelerometer sensor signals.

The DWT adopts binary scales and translations to reduce the amount of computation, which results in better efficiency of calculation [83]. With discrete wavelet transforms, the signals can be broken into custom-sized windows with an assigned resolution for each of the windows. DWT allows analysis of various frequency bands contained within a signal, which could be related to the initial stages of a failure within a structure [70]. In wavelet analysis, DWT divides the signal into approximations and details as outputs. The approximations are the high-scale (amplitude), low-frequency components of the signal. The details are the low-scale, high-frequency components [83]. This process consists of filtering sensor data through a low-pass filter to generate the approximations and a high-pass filter to generate the details.

However, using DWT to process 1000 samples will result in 2000 samples, as the filtering has now doubled the initial set. DWT offers a solution by computing alternations, where one point out of two is kept, and it reduces the output of the approximations and details. The above division of signals into approximations and details is more commonly referred to as the decomposition of a signal [50]. The decomposition can be iterated, with successive approximations being decomposed in turn. The signal is broken into many lower resolution components [82]. The number of decompositions is iterative and, in theory, can continue indefinitely. The number of levels depends on how much low-resolution detail the user requires. In studies using wavelet decomposition in TCM and SHM, the number of decompositions typically lies between 5 and 6 [70,83,84,88].

The decomposition process decomposes the signal and produces threshold levels containing information about damage as captured in the signal. Analysing at a higher level allows data relevant to the tool condition to be decomposed from the signal noise captured by the sensors. The resolution is matched to each decomposition, meaning that there are no losses in the captured information, and any significant transient changes in the tool’s condition can be identified [70,72]. Decomposition of the signal may facilitate the separation of noise frequencies to focus on the waveband associated with a particular defect signature. The higher level of decomposition, the more refined the wavebands.

Beale et al. [89] applied an acoustics-based wavelet packet denoising (WPD) algorithm for structural health monitoring on wind turbine blades. This method offers the best trade-off between signal-to-noise ratio (SNR) improvement and the amount of signal energy retained. This was determined through an intensive computational simulation. Hard thresholding was applied to the acoustic datasets due to their high energy preservation, and the level of decomposition was set at seven iterations as it preserved approximately 90% of the original signal energy. The fine-tuned algorithm improved the detection rate by 60% and reduced the false detection rate by removing both transient and ambient noise from the signal.

Li et al. [85] applied wavelet packet transforms (WPT) to decompose collected AE signals during boring operations. The use of WPT allowed the extraction of features from collected signals related to tool wear states.

Lange et al. [84] used DWT on the ultrasound waves to help determine the ultrasound frequency bandwidth that is most sensitive to tool wear in turning operations. The original signal was decomposed to the fourth level using the Daubechies-4 wavelet filter. The most sensitive response to the tool chatter was located at a high-frequency bandwidth of 8.75–10 MHz at the third level of decomposition.

Zhao et al. [87] used a four-layer decomposition of an AE signal collected from AE sensors mounted on a wind turbine blade. The monitored frequency range was between 0 kHz and 250 kHz. The decomposed signal was divided into 16 bands with a bandwidth of 31.25 kHz. However, only the first eight were monitored due to the defined frequency range.

## 5. Decision-Making Algorithms

Condition monitoring requires automated decision-making to alert machine operators to the fact that a defect has occurred and that corrective action should be taken to rectify the situation. In many cases, condition monitoring occurs in noisy environments where the process or the surrounding environment generates unwanted noise, impacting the collected data. This signal noise can make a failure or defect initiation more challenging to detect, or it can increase the risk of false alarms causing unnecessary delays in production. For this purpose, signal processing techniques covered in Section 4 are implemented to reduce the impact of background noise, enabling the successful application of one or more of the classifier techniques or model-based approaches reviewed in this section. Algorithms for the detection and/or diagnosis of tool defects can be either data-based or model-based.

### 5.1. Data-Based

Data-based approaches capture historical or experimental data representing each possible fault, including tool and machine failures. Such quantities of data typically lead to a high need for computational power for analysing large datasets gathered from the process [90]. These methods have been implemented in various industrial processes and proved capable of generating predictions for wear out of the tool [91].

The data for decision-making algorithms should contain a representation of the healthy state of the tool as well as any potential damage considered to be detectable. For developing a fault classifier using any dataset, the ratio of healthy versus damaged data must be 50:50. If this balance is not maintained, the classifier becomes biased and introduces a need to either oversample or undersample the existing data [92]. The oversampling operation refers to generating artificial samples to balance an unbalanced dataset. Undersampling, on the other hand, refers to removing most of the healthy dataset to match the damaged dataset and reach a 50:50 ratio. There are limitations to both approaches, such as oversampling random copies of faulty samples; this increases the sample size but fails to provide valuable, original information for the model training. Undersampling offers an advantage in terms of computing time but does not actually address the problem of sparse samples. For further details, see the excellent review on breakage monitoring of machine tools by Li et al. [92].

#### 5.1.1. Threshold-Based Detection

The principle of threshold-based detection is based on a set critical value between expected behaviour and the breakage state of the mould tool [92]. This method looks at monitoring the real-time response of the condition monitoring sensors’ characteristics and identifying when the threshold value is exceeded. Exceeding the threshold value triggers an alarm and identifies that damage has occurred. A series of trials generally determine these threshold values by collecting data from the healthy tooling based on the healthy operation of the sensors. This enables an understanding of both the sensor and tooling behaviour in the process environment. With more collected historical data, the set thresholds become more accurate as there is more training data to identify suitable threshold values. Threshold-based detection is applied across machining processes where inputs such as power usage, vibration present and machining time are used to identify the wear of tooling [93].

#### 5.1.2. Artificial Neural Networks

Artificial neural network (ANN) approaches have seen greater uptake in use as decision-making methods, inspired by the working mechanism of the human brain [35]. The ANN model contains three or more layers, each with several neurons represented by an input layer, hidden layer and output layer.

A crucial task in developing neural networks is determining the number of hidden layers and neurons. The inputs to each neuron are weighted. Determining the weights requires training for the best solution. After training, it is expected to obtain the value of the response parameter from the output layer. However, several factors affect the performance of neural networks, such as training time, activation function, weights, and learning function [94]. Several deep learning methods, such as convolutional and recurrent neural networks [95], have been applied recently.

Tool failure prediction using an artificial neural network was performed using measured process variables in a turning process [96]. ANN showed to be 95% and 98% successful at identifying flank wear and crater depth, respectively, in the cutting tools used in a turning process. Kim et al. [97] used ANN successfully for automated crack detection in concrete structures from captured images. The images were captured using a thermal imaging camera and used to classify the structure’s condition based on the collected heat. Yusof et al. [98] implemented convolution neural networks to identify cracks based on RGB images in paved structures with a 94.5% success rate in identifying different types of cracks. Reliable training datasets proved that the network can predict cracking fairly well when tested on unknown datasets.

Hawryluk et al. [99] deployed an ANN-based decision support system model using process parameters and AE readings to analyse the durability of forge tooling used under different operating conditions. They used 450 training cases in this work. The model was developed using data from experiments and computer simulations which were used to train and test the model. As forging is a complex process, the ANN-based system delivered an assessment error of 10%, which was deemed satisfactory. Their work aimed to understand the development of cracks and deformation within the dies. The authors anticipated possible future improvement by connecting it with the process to obtain more extensive training data.

Lange et al. [84] developed a three-layer, multilayer perception artificial neural network using process parameters such as cut depth, cutting speed and output from the DWT of the ultrasound signal. The data points used for training, verification and testing had a 50%/25%/25% split. The training error of the ANN started at 1.4% initially but after 50 iterations the accuracy error levels off at 0.2%.

#### 5.1.3. Support Vector Machine

Support Vector Machine (SVM) is a supervised classification method in machine learning applications. SVM uses a hyperplane to separate data points with a margin represented by so-called support vectors. The exact position and direction of the hyperplane are determined by the support vectors closest to the hyperplane. Specific data points can be removed from the plane to maximise the margin and shape of the hyperplane [100]. SVM uses structural risk minimisation and can therefore handle large data. Chen-Hui et al. [101] performed a simulation using an SVM model to detect delamination size within composite materials using lamb wave signals from piezoelectric transducers. The model effectively identified the magnitude of the delamination in the simulation.

The work of Sari et al. [102] used SVM to detect and classify pavement cracks automatically. The classification was performed on captured images by applying image processing, segmentation and feature extraction to achieve the most accurate prediction. Dib et al. [103] compared SVM for detecting necking (delamination) in sheet metal forming processes. The datasets were created based on numerical simulation results of each sheet metal material used in the U-channel forming process. The data was split into 70% training and 30% validation and compared using multi-layer perception (MLP), random forest (RF), classification and regression tree (CART), naive Bayes (NB) and support vector machine (SVM) using the same number of random weights. After 30 runs, the SVM scored the highest 92.01% success rate at identifying springing (deformation). It is worth mentioning that the MLP score was only 0.12% less effective.

Zhao et al. [87] developed an SVM for identifying crack propagation damage of the blade in a wind turbine using a wavelet decomposed acoustic emission signal. Ten sets of feature vectors were extracted by wavelet packet and a 50/50 split was performed for training and testing of the SVM. By combining the decomposed signal and SVM damage identification method, the crack damage recognition accuracy reached 90% during simulation.

#### 5.1.4. Fuzzy Logic

Fuzzy logic is a well-established decision-making approach that captures incomplete and imprecise information for implementing fairly accurate logic rather than exact logic. Fuzzy logic allows for degrees of membership in a set. The membership of an element to a set is typically rated on a 0–1 scale of possibility. Fuzzification, rule editor and defuzzification are processes commonly applied to input data values [50]. Initially, the range of membership functions is defined by training input values using rules, inputs and outputs linked together. Lastly, fuzzy terms are converted to numerical values using defuzzification.

Kuntoglu and Saglam [104] used AE and tool tip temperature data, in addition to process parameters, as inputs to a fuzzy interference model for predicting the chipping of the cutting tool with a high success rate. Gowd et al. [105] compared fuzzy logic (FL) and ANN for crack detection in beam structures. The experimental work was conducted using a simulation of varying crack depths and locations. In each algorithm, three relative frequencies were used as inputs, with crack location and depth as the two outputs. Additionally, fuzzy logic was based on triangular, trapezoidal and Gaussian membership functions with the same setup of three inputs and two outputs. Gaussian fuzzy logic performed better at determining relative crack depth (98.7% vs. 98.3%), and ANN performed better at determining relative crack location (92% vs. 88.4%).

### 5.2. Model-Based

**Figure 10:**
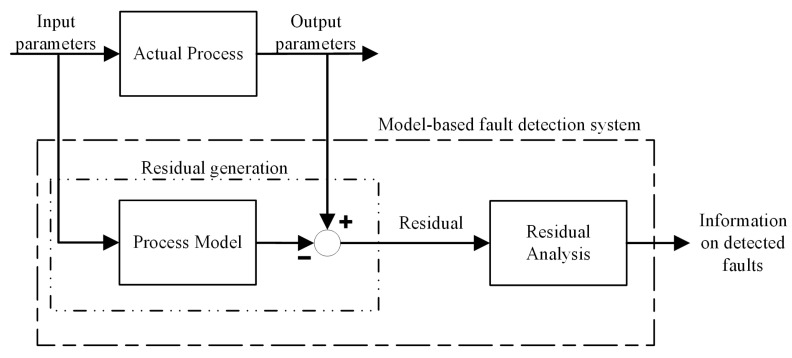
Representation of an observer-based fault diagnosis system.

Model-based approaches are deployed in processes where real-time monitoring is possible and where a model of the system outputs (the monitored variables) based on inputs can be defined to generate an observer. The model is used to predict the signal output under normal healthy operation, and by comparison of the residuals between the actual signal output and the model output, a fault can be identified [106]. Figure 10 illustrates a model-based system for fault detection.

Real-time sensor data are required to successfully implement an observer model into TCM. If the system is observable and the process parameters are known, then the output of the tool or structure can be estimated by the deployed observer. The residuals between the modelled and measured data can be analysed to determine if a fault has occurred. Various fault detection and diagnosis observers have been proposed in the literature [107]. However, an accurate and complete mathematical model of a process is rarely achievable in practice. Therefore, model uncertainties need to be considered, which can affect the generation of residuals [108]. The most popular approaches are based on the Kalman filter (KF), which models the uncertainty in the process model and the measurements and uses Bayesian inference to determine the optimum estimate of the states [109]. The H∞-based observer enables the incorporation of frequency specifications as additional criteria for better fault discrimination [110]. Model uncertainties can be influenced by disturbances representing unknown or uncontrolled inputs acting on the system. The use of an adaptive observer has been proposed, which can be applied to linear and nonlinear systems to overcome this issue [111].

Model-based methods are applied in machining for tool wear monitoring, where the tool wear can be modelled as a simple linear progression over time and combined with noisy measurements from, e.g., vision systems. Model-based methods are also commonly applied in structural health monitoring, for example, in aerospace and wind turbine structures where the vibrations of the healthy structure under loading can be predicted by dynamic models [111,112]. However, the application of model-based techniques has not been extended to tool breakage problems, as it is very difficult to predict AE or vibration signals in a noisy machine environment. For this reason, the potential of model-based methods is also more limited in the injection moulding environment.

However, Chen et al. [24] used a mathematical model based on an extended Kalman filter to detect the presence of contaminations (impurities) of material or second-phase precipitations in the mould tool. They used the input data provided by process parameters such as servo system data containing speed, position and current usage. The proposed model showed great potential in identifying foreign bodies and avoiding deformation within mould tools. The model was validated in a process setting and showed that the detection distance of foreign bodies was decreased by 22%, detecting foreign bodies sooner than the standard built-in mould protection function in the IMM machine used. Table 1 shows a comparison overview between data-driven and model-based approaches for defect detection.

### 5.3. Summary

Signal processing and decision-making algorithms presented in Section 4 and Section 5 demonstrate their capability in identifying defects such as cracking and delamination based on sensor data, including the ability to monitor the propagation of defects as they become more severe. Approaches such as Fourier transforms, wavelet analysis, machine learning classifiers, and model-based observers have been applied in several industries for SHM and TCM, including aerospace and wind turbines but also in other forming processes using dies and moulds.

## 6. Discussion

The high clamping forces, high temperatures and the potential for cooling channel blockages, combined with high volume production cycles, all contribute to injection moulding being a high-risk environment for tooling damage and even catastrophic failures. The occurrence of tool failures is historically rare for conventional tooling, but defects such as deformation and cracking have been known to occur within the injection moulding industry. If a failure occurs, it can cause significant machine damage, resulting in high repair costs.

The introduction of AM tooling to IM processes further exacerbates the risk by introducing other tooling defects and failure modes, such as cracking due to porosity, or delamination due to improper printing, as reviewed in Section 2.1 and Section 2.2. The increased risk and lack of historical confidence in AM tooling in injection moulding leave uncertainty within the industry regarding the introduction of AM tooling to their processes.

The IM process to date has not seen much research activity in the application of tool condition monitoring (TCM). However, this is expected to be a necessary enabler for the uptake of AM tooling into injection moulding processes. Introducing AM technology into tool manufacturing can bring significant benefits to the IM process. However, confidence in tooling longevity still needs to be reinforced. To provide this confidence, there is a need within the IM industry to deploy TCM in mould tooling, not only for AM tools but for standard tools also. Applying TCM to injection moulding can make the process more reliable when working with high production volumes and short lead times.

This review highlights some of the sensor technologies with proven applications in implementing TCM systems. Sensors such as acoustic emission, accelerometer and ultrasound have been shown to be very effective at detecting defects such as cracking, delamination, channel blockages and deformation, all of which are common to IM, AM and other industrial processes. The use of TCM and structural health monitoring (SHM) has proven to be effective and reliable in industries such as machining [37], aerospace [113] and wind turbines [39] in identifying the aforementioned types of defects.

Users must be mindful of critical considerations such as the available installation space, proximity to the region of interest, and the probe specifications such as frequency and signal processing requirements; whereas many commercially available probes are bulky, there have been technological advances in sensing technology and there is future potential for integration of miniaturised sensors, i.e., microelectromechanical systems (MEMS) with wireless communication to avoid the need for cable channels through the mould tool. Miniature and wireless sensors could potentially be embedded into the tool during the 3D-printing process as presented by Tomaz et al. [114]. However, concerns remain regarding the transmission of wireless signals and power delivery through stainless steel [115]. Embedded sensors provide customisation and placement at desired locations through additive manufacturing [116]. However, issues remain with later accessibility to the embedded sensors which lead to complex repairs of the sensors. Ideally, the sensor lifespan would match that of the tool, however, given the harsh machine environment this is currently a challenge for many types of sensors.

In some cases, defects can be detected using basic limit thresholds on selected features of the sensor signals. Defects such as delamination and cracking impact the original structure of the tool. With sensors such as acoustic emission and accelerometers, the generated acoustic waves and vibrations will then typically fall outside of the originally defined limits. However, if the sensors are used in a noisy environment, further signal processing is needed to separate signal features associated with tool faults from signals associated with other sources. Signal processing methods, including Fourier or wavelet transforms, enable the extraction of specific frequency ranges of relevance to defect initiation and use these as inputs for threshold analysis or more sophisticated algorithms for the classification of tool health.

The decision-making algorithms can be grouped into two categories, data and model approaches. Data-based fault detection methods have received much attention from industries for both process monitoring [117,118,119] and tool condition monitoring [120].

The main issue associated with data-driven approaches is the requirement for large quantities of historical, verified data to provide reliable performance [121]. Data-driven approaches require a large set of sensor data for each type of fault to train the model before the system’s deployment. It is time-consuming and expensive to generate a sufficient volume of fault data for data-driven approaches, and the collected data are often unique to the tooling being monitored. The collection of large quantities of data for specific defects requires the formation of data through destructive testing, where defects are created and tested, and data are collected at the expense of manufacturing several test samples.

In comparison, model-driven algorithms first require a physical model of the process, which needs to be sufficiently accurate to create a process benchmark. The challenge remains in accurately modelling the healthy behaviour of sensors, especially in noisy processes. Model-based approaches compare the real-time sensor data readings against the model to identify if the process has diverged from a normal, healthy operation. This can alert end users to tooling and sensor anomalies and potential risks to the process. Difficulties in modelling AE and accelerometer outputs in a noisy machine environment have so far limited the application of model-based approaches to monitoring the progression of tool wear in machine tools, rather than detection of the onset of tool breakages. However, the modelling of sensor behaviour is complex and time-consuming, especially within a multi-sensor TCM that is monitoring for several defect types.

As presented by Chen et al. [24], model-based approaches have been applied in injection moulding for foreign body detection using the IM process variables as inputs. As foreign bodies trapped between the mould halves can result in damage to the tool, such a system can help prevent tool damage before it occurs. However, not all damage can be prevented by monitoring process variables, enforcing the need for TCM systems that detect tool defects upon occurrence in AM and conventional injection mould tooling.

Based on the reviewed research works, TCM systems can help achieve more sustainable manufacturing processes by preventing, and in some instances predicting, tooling failures which serve to minimise repair costs, scrap generation and process downtime.

The development of a tool condition monitoring (TCM) system for additively manufactured injection mould tools would enable the industry to yield the promised benefits of AM tooling, including greater design freedom and significantly shorter cycle times afforded by conformal cooling. For the tool-making industry, TCM systems provide a foundation for the broader adaptation of AM tooling that operates under the digital principles of Industry 4.0 [16].

Applying the principles of TCM to AM tooling in injection moulding also opens up opportunities for new business models founded in leasing tooling to end users. Such business models could benefit both the customer and manufacturer by offering access to real-time data from the tool leading to greater process control. Such a data-driven service for tools and process monitoring can give insights into the tool performance/design for continuous improvement initiatives and also offer opportunities to implement preventative maintenance strategies.

## 7. Conclusions

The use of additively manufactured injection mould tooling offers a range of benefits not limited to improved cycle times and optimised component quality attributes. Introducing tool condition monitoring into injection moulding (IM) would accelerate the adoption of additively manufactured (AM) cavity tooling and the benefits it brings to the process by instilling industry confidence. To detect defects common to IM and AM processes, the most promising sensors include acoustic emission, accelerometers and ultrasound sensors. These sensing technologies show the greatest potential for detecting the potential defects of cracking, delamination and cooling channel blockages which are a concern for AM tooling, and are feasible for incorporation into a mould tool. Each of the sensors has their own advantages and limitations in detecting different faults and the integration of all three technologies show greatest potential for identifying multiple sources of tool condition problems.

Due to large amounts of machine noise present in injection moulding, signal processing strategies, namely time–frequency domain methods such as short-time Fourier-transform (STFT) or discrete wavelet transform (DWT), are powerful methods for the transformation of the raw sensor data to a form more amenable for the detection of changes in tool condition. Each of the strategies offers different advantages depending on the sensor data; both STFT and WDT should be implemented into the design and validated for best performance.

Data-based approaches for the classification of tool health are perceived as the best contenders for use in injection moulding when using multiple sensors. Popular data-based approaches include artificial neural networks (ANN), support vector machine (SVM) and fuzzy logic, which all present suitable capabilities and have been successful for condition monitoring in other industries, including machining, wind turbines and structural bridges.

Future research should focus on identifying and testing suitable algorithms for fault detection using multi-sensor input data for the development of smart additively manufactured tooling in the injection moulding industry. The collected data and proposed tool condition monitoring system can be used as the basis for building datasets for artificial intelligence strategies which can be implemented in the injection moulding process.

## Figures and Tables

**Table 1:** Pros and cons of data-driven and model-driven TCM approaches.

	Pros	Cons
Model-based	Does not require historical data for generation of physical model of the system Offers identification of faulty sensors based on generated residuals Faulty behaviour is monitored based on generated signature matrix through structural analysis	Such systems require high computational power Difficult to model response of AE and accelerometer sensors in the complex machine environment
Data-based	This approach does not require high computational power Detects fault based on sensor output and comparison to the collected healthy dataset	Requires a large amount of historical data about the system Sensor fault is more difficult to identify

## Data Availability

Not applicable.

## Abbreviations

The following abbreviations are used in this manuscript:

AE	Acoustic Emission
AM	Additive Manufacturing
ANN	Artificial Neural Network
CART	Classification Furthermore, Regression Tree
CCC	Conformal Cooling Channels
CNC	Computer Numerical Control
CNN	Convolution Neural Network
CWT	Continuous Wavelet Transform
DWT	Discrete Wavelet Transform
EDM	Electrical Discharge Machining
EKF	Extended Kalman Filter
FL	Fuzzy Logic
FT	Fourier Transform
IM	Injection Moulding
IMF	Intrinsic Mode Function
IMM	Injection Moulding Machine
KF	Kalman Filter
MEMS	Microelectromechanical systems
MLP	Multilayer Perception
MODBNE	Multiobjective Deep Belief Networks Ensemble
MSE	Multiscale Entropy
NB	Naive Bayes
NDT	Nondestructive Testing
RF	Random Forest
RMS	Root Mean Squared
SHM	Structural Health Monitoring
STFT	Short-Time Fourier Transform
SVM	Support Vector Machine
TCM	Tool Condition Monitoring
UKF	Unscented Kalman Filter
WPD	Wavelet Packet Denoising
WPT	Wavelet Packet Transform
